# Antibiofilm Properties of Miswak (*Salvadora persica*) Compared to Conventional Oral Health Measures: A Systematic Review

**DOI:** 10.1155/sci5/9344221

**Published:** 2025-05-29

**Authors:** Ipsitha Vejendla, Jogikalmat Krithikadatta, Thilla Sekar Vinothkumar, Kumar Chandan Srivastava, Balqees Mohammad Dhuwayhi Alruwaili, Manal Yahya Saleh Aljarid, Deepti Shrivastava

**Affiliations:** ^1^Department of Conservative Dentistry and Endodontics, Saveetha Dental College and Hospitals, Saveetha Institute of Medical and Technical Sciences (SIMATS), Saveetha University, Chennai 600077, Tamil Nadu, India; ^2^Department of Cariology, Saveetha Dental College and Hospitals, Saveetha Institute of Medical and Technical Sciences (SIMATS), Saveetha University, Chennai 600077, Tamil Nadu, India; ^3^Department of Restorative Dental Sciences, Division of Operative Dentistry, College of Dentistry, Jazan University, Jazan 45142, Saudi Arabia; ^4^Oral Medicine & Radiology, Department of Oral & Maxillofacial Surgery & Diagnostic Sciences, College of Dentistry, Jouf University, Sakaka 72345, Saudi Arabia; ^5^College of Dentistry, Jouf University, Sakaka 72345, Saudi Arabia; ^6^Department of Preventive Dentistry, College of Dentistry, Jouf University, Sakaka 72345, Saudi Arabia; ^7^Department of Periodontics, Saveetha Dental College, Saveetha Institute of Medical and Technical Sciences (SIMATS), Saveetha University, Chennai 600077, India

**Keywords:** bacterial count, dental caries, dental plaque, herbal, miswak, *Salvadora persica*, tooth brushing

## Abstract

**Aim:** The systematic review aim was to assess the antibiofilm properties of miswak (*Salvadora persica*) in comparison with conventional oral health measures in the adult population.

**Materials and Methods:** A comprehensive search strategy was formed, and electronic databases (Scopus, PubMed, Web of Science, ProQuest, and Cochrane Library), gray literature, and clinical trials registry (clinicaltrials.gov) were searched to identify the relevant articles. The inclusion criteria were based on the PICOS strategy. Randomized controlled trials (RCTs) involving the adult population comparing the use of miswak with conventional oral health measures were included in the review. Adult populations undergoing orthodontic treatment and other study designs were excluded. The included studies were assessed for qualitative analysis, and the data extraction was done by two independent reviewers. The outcome of the review was to compare the antibiofilm activity of miswak and conventional oral hygiene measures. The risk of bias in these research studies has been determined based on the guidelines of the Cochrane Handbook for Systematic Reviews of Interventions.

**Results:** Out of 474 identified articles, 10 studies have been included for qualitative analysis, based on eligibility criteria, with a total of 442 individuals. These studies, published between 2011 and 2023, primarily focused on assessing the antiplaque efficacy of miswak and its effectiveness in reducing cariogenic bacterial load. Miswak exhibited positive effects in antibacterial and antiplaque activity, showing the potential to reduce plaque accumulation and gingival inflammation.

**Conclusion:** When *Salvadora persica* is used to maintain dental hygiene, there is a noteworthy decrease in the amount of cariogenic bacteria and plaque. To evaluate the benefits and appropriate outcomes of miswak practice in terms of maintaining dental health, more clinical research is advised.

## 1. Introduction

Oral hygiene is a critical aspect of maintaining overall health and well-being, as it directly influences the integrity of the oral cavity and has far-reaching implications for systemic health [1]. Conventional oral hygiene measures, such as toothbrushing, flossing, and mouth rinsing, have long been advocated as essential practices to maintain optimal oral health [2, 3].

The use of organic resources as medicines has been reported in the form of traditional drugs, therapies, potions, and oils. Most of them contain bioactive natural substances that remain unidentified [4]. Exploring natural products and alternative methods to improve oral hygiene has gained popularity in recent years. Herbal products have been utilized to prevent diseases and enhance well-being since ancient times [5, 6]. Naturally derived compounds possess diverse chemical structures with high biochemical specificity and favorable molecular properties that are desirable for drug discovery and development [7]. The majority of these compounds are plant-based secondary metabolites, such as flavonoids, phenolic acids, tannins, anthraquinones, alkaloids, terpenoids, and stilbenes. These compounds effectively inhibit the growth of pathogenic microorganisms [8, 9]. One such alternative is to employ *Salvadora persica*, commonly known as miswak, a traditional oral hygiene tool with a rich historical and cultural background. The practice of using chewing sticks derived from this plant for oral hygiene is widely recognized in traditional Arabic culture [10]. For about seven thousand years, the Babylonians instigated the use of these chewing sticks for dental hygiene. This practice spread to various cultures including Egypt, Rome, Greece, and the Islamic kingdoms, and persists in many regions globally, notably in Asia, Africa, South America, and the Middle East countries such as Saudi Arabia and other Islamic countries [11]. The World Health Organization has recommended and endorsed the use of chewing sticks made from *S. persica* as an effective tool for oral hygiene in regions where this practice is customary [12].

*Salvadora persica* L (“toothbrush tree”), which is a *Salvadoraceae* family member, is widely used in many Middle Eastern, Asian, and African countries [13], as chewing sticks, to maintain oral hygiene [14]. Miswak has its special features for use, wherein a thin piece of bark is stripped off at the useful end and then chewed. When miswak is chewed, fibers are frayed, giving it a brush-like look that makes it easier to clean teeth [15]. The mechanical cleansing action of miswak contributes to its oral hygiene benefits. The natural bristles of the miswak stick help remove food debris, stains, and plaque from the teeth, promoting oral cleanliness [16].

The antimicrobial activity of miswak has been extensively studied and attributed to its active constituents, including alkaloids, flavonoids, and essential oils. According to the reviews, the extract of *Salvadora persica* contains a wide range of organic (such as saponins, glycoside, alkaloids, benzyl derivatives, flavonoids, tannins, phenolic compounds, and organic acids) and inorganic substances (such as chloride, fluoride, thiocyanate, nitrate, and sulfate) [17]. Miswak possesses the ability to impede the growth of microbes that cause periodontal disease [18–22]. Previous literature reports that miswak has significant antimicrobial activity against *Actinobacillus, Streptococcus mutans, Porphyromonas gingivalis, Enterococcus faecalis, Haemophilus influenza,* and to a lesser extent, against *Lactobacillus species* [23]. Sofrata et al. in 2011 identified benzyl isothiocyanate in the extracts of *Salvadora persica* and demonstrated its rapid and potent bactericidal effect against Gram-negative bacteria [24].

While conventional oral hygiene measures such as tooth brushing and flossing are widely practiced, miswak as an alternative oral hygiene tool has gained attention due to its natural origin and potential additional benefits [25]. Aqueous miswak extract demonstrated antimicrobial effects, in in vitro studies, against specific oral bacteria, such as *S. mutans, Staphylococcus aureus*, and anaerobic species of *Streptococcus*, with growth inhibition levels depending on the concentration of the extract [26, 27].

With growing interest in natural alternatives, it is important to compare the efficacy and advantages of miswak with conventional oral hygiene measures to provide evidence-based recommendations for individuals seeking alternative oral hygiene options. The following systematic review is conducted to determine the efficacy of miswak (in all forms) to be used as an alternative and/or adjunct to the oral hygiene practices that are followed daily. Therefore, this systematic review aims to assess the antibiofilm properties of miswak (*Salvadora persica*) in comparison with conventional oral health measures in adult permanent teeth. Based on the PICOS (Population, Intervention, Comparison, and Outcome, Study design) strategy, the research question was formulated [28]: In randomized controlled trials (RCTs) (S) involving adult patients (P), is the use of miswak (*Salvadora persica*) (I) superior to or comparable with conventional oral health measures (C) in terms of antibiofilm activity (O)?

## 2. Materials and Methods

### 2.1. Review Registration

This systematic review was registered on PROSPERO (http://www.crd.york.ac.uk/prospero/) with registration number CRD42023439444. The review protocol was designed following the PRISMA (Preferred Reporting Items for Systematic Review and Meta-Analyses) 2020 guidelines.

### 2.2. Search Strategy

This comprehensive systematic review of RCTs compared the use of *Salvadora persica* to standard oral health maintenance measures, in terms of its antibiofilm activity. A search strategy was made, and electronic databases, registers, and gray literature were searched to identify the relevant articles. The databases evaluated were Scopus, PubMed, ProQuest, Web of Science, and Cochrane Library, along with the clinical trials registry (clinicaltrials.gov). The search strategy included relevant keywords (MeSH terms) and Boolean operators, with MeSH terms appearing in the title and abstract up to July 31, 2023. There have been no restrictions in terms of language. The search strategy performed for all the electronic databases is listed in Table 1.

### 2.3. Eligibility Criteria

The papers have been screened as per the PICOS strategy and the following inclusion criteria were formulated:

• Population: Adult patients (above 18 years of age)
• Intervention: All forms of *Salvadora persica* (miswak)
• Comparison: Standard oral health measures (toothbrushing, mouth rinse, fluoride, flossing, and interdental aids)
• Outcomes: Antibiofilm activity that could be expressed as
  • Antiplaque activity (plaque indices)
  • Colony forming units (CFU) count in plaque and saliva (total bacterial count and total microbial count)
  • Glucosyltransferase activity in saliva and plaque determines the enzyme activity (in terms of OD values)
  • Assays like crystal violet staining and confocal laser scanning microscopy (CLSM) to determine the number of viable cells
  • Anticaries activity—measured using Missing, Decayed, and Filled Teeth (DMFT), Decayed, Missing, and Filled Surfaces (DMFS), International Caries Detection and Assessment System (ICDAS), or any other caries-detecting index employed in the conducted trial that determines the past and present caries experience
• Study selection: RCTs

The exclusion criteria have been as follows:

• Other study designs such as case series/reports, literature reviews, case-control studies, cohort studies, in vitro studies, in vivo studies, cross-sectional studies, and animal studies were excluded.
• Individuals with orthodontic wires/brackets.
• Studies done other than *Salvadora persica.*

### 2.4. Screening Process

The screening for the eligible studies was done using the Rayyan software 9. In the initial phase, the two reviewers independently searched the databases, and based on the titles and abstracts, each study was categorized as either eligible, not eligible, or potentially eligible. The eligible articles were shortlisted for inclusion in this review, followed by retrieval of the full-text publication. The corresponding author would be promptly contacted in the event of any identified missing data within the studies under review. Any of the uncertainty as well as disputes among the reviewers has been resolved through consensus in the existence of a third reviewer.

### 2.5. Data Extraction

One reviewer obtained the data from the included studies, and the second author then independently evaluated the data to make sure it was accurate and relevant. The third reviewer resolved any discrepancies. The following data were analyzed:

• The design and location of the study.
• Total sample size, distribution of samples among groups, and demographic characteristics of the participants.
• The form of *Salvadora persica* employed in the study.
• The frequency of the interventions.
• Clinical parameters assessing the outcome.
• Duration of follow-up intervals.

The included papers were thoroughly reviewed to find similarities for conducting a meta-analysis. However, due to the heterogeneity in trial duration and a limited number of studies, performing a meta-analysis was not feasible.

### 2.6. Risk of Bias Assessment

The risk of bias evaluation followed the guidelines in the Cochrane Handbook for Systematic Reviews of Interventions [29]. The included studies were evaluated across six main domains: performance bias, detection bias, selection bias, reporting bias, attrition bias, and other potential sources of bias. The following judgments were made: low risk, unclear risk, or high risk. Both reviewers independently conducted the assessment for each included study, with any discrepancies resolved through discussion with the third reviewer.

## 3. Results

### 3.1. Study Selection

**Figure 1:**
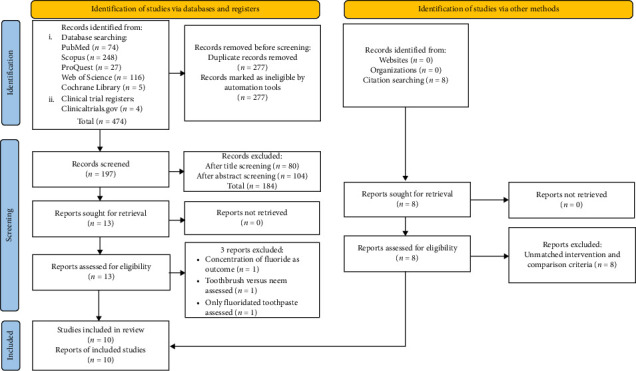
PRISMA flowchart.

Figure 1 illustrates the outcomes of the search strategy. 474 potentially eligible articles have been identified published from inception till July 31, 2023. The distribution of results across the five databases is as follows: PubMed (74), Scopus (248), Web of Science (116), ProQuest [27], and Cochrane Central Register of Controlled Trials [5], and 4 studies retrieved from the trial registry (clinicaltrials.gov). 277 of these were eliminated as duplicates. The remaining 197 articles were assessed based on their titles and abstracts. After the titles and abstracts were evaluated, 184 articles were eliminated as they did not meet the predetermined inclusion criteria. The 13 remaining papers were thoroughly examined in their entirety in order to ensure that they met the preset inclusion requirements. As a result, three studies that did not fit the inclusion criteria have been eliminated. The reasons for exclusion are listed in Table 2. Overall, 10 studies were considered for the qualitative analysis. The kappa coefficient revealed a 100% agreement between the reviewers in their assessment including search strategy, screening, and data extraction of the included articles. A meta-analysis could not be performed for this systematic review due to critical limitations related to methodological variability and data heterogeneity. The differences in sample size, intervention protocols, outcome measures, and follow-up periods account for significant heterogeneity and lack of standardization that prevents meaningful statistical pooling of data. Several studies provided insufficient statistical data, making data extraction for meta-analysis unfeasible.

### 3.2. Summary of Characteristics of Included Studies

Table 3 enlists the features of all the included studies. In this review of 10 included studies investigating the impact of miswak on dental health, diverse populations from different locations were studied. All 10 studies included were RCTs, conducted in Saudi Arabia, Iraq, India, Iran, and Egypt. A total of 442 participants were assessed, of which 199 were males and 213 were females. All the participants in the studies were aged between 18 and 26 years. The majority of the studies used miswak in the form of chewing sticks for assessment of dental health. The included studies assessed the antiplaque, antibacterial, anticariogenic, and antigingivitis activity of miswak.

### 3.3. Assessment of Antiplaque Activity

Five studies evaluated the antiplaque effectiveness of miswak using the Modified Quigley–Hein and Loe–Silness plaque indices, demonstrating its positive impact on controlling dental plaque. Al-Otaibi et al. reported a significant reduction in plaque accumulation and gingivitis, particularly in maintaining approximal gingival health [30]. Zarabadipour et al. and Sofrata et al. compared active and inactive miswak with standard oral hygiene practices and suggested miswak as an effective mechanical toothbrushing aid, though no significant difference was observed between active and inactive miswak in reducing approximal plaque and subgingival microbiota, indicating that its mechanical action is responsible for the antiplaque effect [32, 36]. Patel et al. supported these findings, emphasizing that miswak's efficacy in plaque control improves when used alongside regular toothbrushing [33]. Taha et al. showed significant reduction in plaque index over time within each group; however, no statistically significant differences were found when comparing the groups (standard oral hygiene, miswak alone, and miswak with a toothbrush and toothpaste). Risk ratio analysis further indicated that miswak's effectiveness was comparable to other oral hygiene methods, suggesting its potential as an alternative or adjunctive tool for plaque control. The study recommended extended follow-up periods to further validate these findings [37]. Overall, these studies highlight miswak as an effective mechanical aid for plaque control, with additional benefits when combined with conventional toothbrushing.

### 3.4. Assessment of Bacterial Count

Four studies evaluated the antibacterial efficacy of miswak in reducing *Streptococcus mutans* CFU in saliva [34, 35, 38, 39]. Al Dabbagh et al. found that miswak toothpaste and mouthwash significantly reduced *S. mutans* and *Lactobacilli* counts over 2 weeks compared to ordinary toothpaste and saline [34]. Raina et al. reported that miswak sticks impregnated with 0.5% sodium fluoride were as effective as plain miswak sticks in reducing *S. mutans* levels [35]. Kengadaran et al. compared miswak toothpaste with homeopathic and fluoride toothpaste, finding similar reductions in bacterial counts, though miswak toothpaste led to a slight, statistically insignificant increase in plaque pH over 28 days, suggesting potential anticariogenic effects [39]. Shaalan et al. compared miswak herbal toothpaste with fluoride toothpaste in high-caries-risk patients, showing a similar reduction in *S. mutans*, though fluoride toothpaste had greater remineralization potential due to higher fluoride, silicon, phosphorus, and calcium ion release [38]. Additionally, Al-Otaibi et al. assessed miswak's impact on subgingival microbiota, revealing significant microbial reduction comparable to toothbrush use, with a greater decrease in *Actinobacillus actinomycetemcomitans* levels [31]. Collectively, these studies suggest that miswak demonstrates antibacterial efficacy similar to conventional oral hygiene measures, with potential additional benefits in modifying plaque pH and selectively targeting specific bacteria.

### 3.5. Assessment of Anticaries Activity

Only one study assessed the anticaries activity of miswak compared to standard oral hygiene measures. Rashad Taha et al. evaluated the DMFT score, comparing the use of miswak and conventional oral hygiene practices, for over 1 year [37]. The study found that both miswak and standard preventive measures significantly reduced caries in young Egyptian adults, with no notable differences between the groups. Due to the lack of additional studies assessing caries activity, other indices could not be evaluated, limiting the ability to draw comprehensive conclusions about miswak's effectiveness in caries prevention. Combination of other indices (such as DMFS and ICDAS) allows to identify early lesions and lesion progression that allows better treatment planning and caries prevention [40].

### 3.6. Assessment of Gingival Status

The assessment of gingival inflammation was reported in terms of the modified gingival index and Loe–Silness gingival index. Sofrata et al. investigated the impact of active and inactivated miswak on gingival inflammation and plaque accumulation in gingivitis patients. Their findings indicated that fresh miswak did not outperform inactivated miswak in interproximal embrasures and may have had a limited impact on the study population [32]. Al-Otaibi et al. and Patel et al. demonstrated significant reduction in gingivitis when miswak was used along with toothbrushing [30, 33].

### 3.7. Risk of Bias Assessment

**Figure 2:**
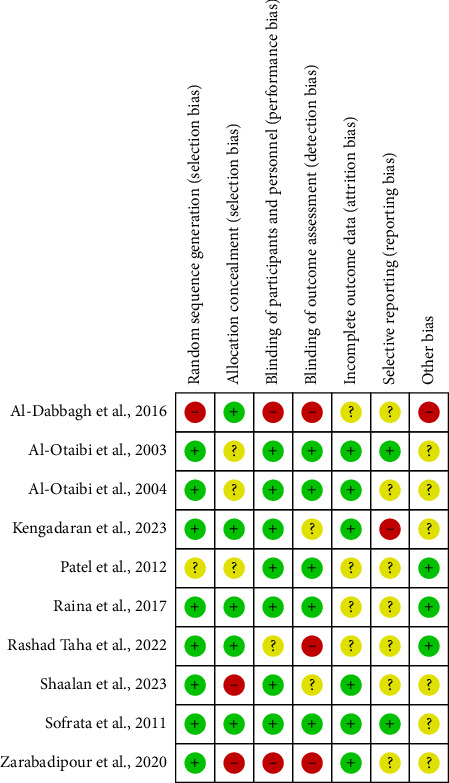
Risk of bias (ROB) for the studies included in the systematic review.

**Figure 3:**
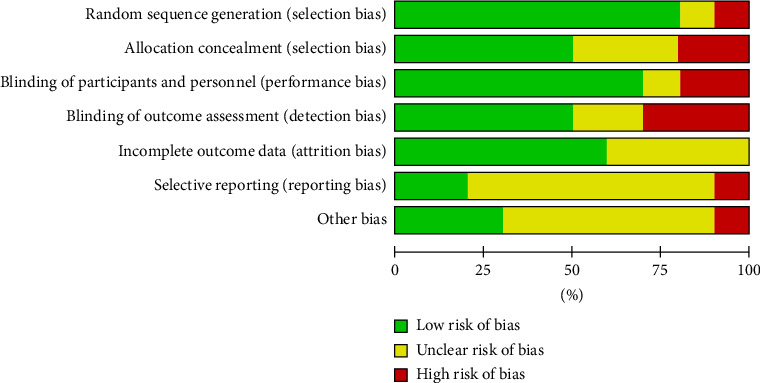
Risk of bias graph.

Figures 2 and 3 summarize the risk of bias assessment of the included studies. Four studies satisfy the majority of domains of risk assessment, suggesting a low risk of bias. The majority of studies report unclear risk with attrition and reporting bias.

## 4. Discussion

With the evolution of dentistry, the emphasis is shifting increasingly toward preventive procedures that focus on the root causes of oral disease [41]. Throughout history, natural remedies have been integral to traditional medicine. Within dentistry, phytomedicines have gained extensive utilization. Medicinal herbs offer valuable and efficient resources for addressing diverse diseases [42]. Topical application of herbal extracts, such as *chamomile, Ocimum (basil), and Echinacea*, has been found to offer therapeutic advantages within the oral cavity. These natural extracts, when applied directly to oral tissues, have demonstrated notable beneficial effects [43, 44].

This systematic review assessed the efficiency of the most widely used herbal plant, *Salvadora persica* (commonly known as miswak), in terms of inhibiting biofilm formation by cariogenic bacteria. *Salvadora persica*, commonly referred to as miswak, belongs to the *Salvadoraceae* plant family [13]. It predominantly thrives in subtropical and arid regions spanning the Middle East, Africa, and the Indian subcontinent [13, 45]. This plant's fresh leaves, twigs, and roots have found applications both in daily dietary practices and traditional herbal therapy [13, 46].

Phytotherapeutic antimicrobial agents offer a potential alternative to synthetic compounds, considering the potential negative effects associated with the latter [47]. Several studies have underscored the variable antimicrobial activity of herbal-based toothpaste [12, 27, 48–51]. The investigations conducted by Mohammed [52] and Adwan et al. [53] yielded consistent findings, indicating the antimicrobial efficacy of toothpastes with miswak extract against various microorganisms. These outcomes are aligned with the findings synthesized in this comprehensive review [34, 35].

Standard oral hygiene practices, encompassing toothbrushing, flossing, and mouth rinses, primarily rely on mechanical actions to remove dental plaque and debris. The favorable attributes of miswak concerning dental and oral well-being arise from a dual mechanism. Firstly, its mechanical utility in brushing augments its beneficial effects. Secondly, its pharmacologically active components, including tannins that hinder glucosyltransferase enzyme activity, thereby reducing plaque and periodontal concerns, and resins that act as a defense against dental caries, contribute to its oral health merits [54]. Two randomized trials conducted in Iraq and Egypt, that compared the effects of miswak with regular toothbrushing, demonstrated miswak's potential for plaque reduction and its noninferiority compared to conventional methods. The studies suggested that miswak-based products exhibit antimicrobial properties, offering benefits beyond mechanical cleaning. These findings align with miswak's bioactive components, potentially enhancing its appeal as a holistic dental care tool [34, 37].

Scientific investigation is drawn to the concept of fluoridating miswak chewing sticks to combine the mechanical cleaning with the anticariogenic impact brought about by fluoride release into the oral cavity. According to Raina et al., plain miswak sticks and those impregnated with sodium fluoride were equally effective at lowering the levels of *Streptococcus mutans* in saliva. These findings are consistent with a RCT carried out in 2014 by Yavagal et al., which found that fluoridated miswak sticks could lower salivary *S. mutans* counts but not *Lactobacillus* counts [35].

Assays such as crystal violet staining and CLSM were utilized to assess the antibiofilm activity of miswak. These techniques provide valuable insights into the structural and microbial composition of biofilms. However, the number of studies utilizing these assays was limited, warranting further investigation to explore the impacts of miswak on biofilm formation as well as organization.

Regarding the anticaries activity, the studies primarily utilized clinical parameters such as the DMFS index, DMFT index, or ICDAS. However, the valuation of caries activity varied across the studies, making it challenging to draw definitive conclusions. The study by Taha et al. found that both miswak and standard preventive measures had a significant impact on reducing caries in young Egyptian adults, with no major variation between the 2 groups, indicating a potential reduction in caries activity with the use of miswak that could be comparable to conventional oral health measures [37]. The majority of the available literature supports the use of miswak in reducing caries incidence in the young population as well as adults [12, 27, 49]. However, further research with standardized and consistent caries assessment methods that could determine the caries activity in terms of indices and microbiological assay is necessary.

The long-term efficacy of miswak in maintaining oral health remains an area requiring further investigation. While several short-term studies have demonstrated its antibacterial effects, limited data exist on its sustained impact over extended periods. Factors such as frequency and technique of use, individual oral hygiene habits, and variations in miswak preparation may influence its long-term effectiveness. Evaluating miswak's durability in maintaining plaque control, its ability to prevent biofilm maturation, and its long-term impact on overall oral microbiota composition would provide deeper insights into its effectiveness as a sustainable oral hygiene tool. Although our systematic review offers insightful comparisons between miswak and conventional oral hygiene practices, it is vital to recognize some limitations that might have affected how our results should be interpreted and extrapolated. The evaluated studies are subject to several potential limitations. A subset of these studies featured relatively small sample sizes that could impede the generalizability of their results. Additionally, certain investigations employed interventions of short duration, ranging from days to weeks, limiting insights into the potential long-term effects. Another noteworthy limitation was the absence of participant blinding in certain cases, which could introduce bias into the results. The heterogeneity of interventions across studies poses challenges in drawing cohesive conclusions. Moreover, the lack of follow-up in certain studies restricts the understanding of sustained intervention impacts. Miswak's cultural importance may alter depending on the population, and cultural practices may have an impact on how it is used. The differences in oral habits, diet, oral microbiome, and compliance to the herbal treatment play a major role. The frequency and greater adherence to the use of miswak by traditional users may not be replicated by the nontraditional population, which highly impacts generalizability and study outcome. The applicability of our findings to other geographical areas may be impacted by this issue. These limitations collectively underscore the need for cautious interpretation of results and emphasize the importance of addressing these issues in future research endeavors.

### 4.1. Clinical Relevance

The exploration of miswak holds significant promise for future research and practical applications within the field of oral health. Deeper investigations into the mechanisms behind antimicrobial, anti-inflammatory, and biofilm-inhibiting properties of miswak are warranted [11, 55]. Future research could focus on investigating specific bioactive compounds responsible for therapeutic effects and their interactions with oral microorganisms, thereby aiding in development of targeted interventions for oral health maintenance and disease prevention. As the world becomes more environmentally conscious, the use of natural products like miswak gains relevance. Assessing the ecological impact of widespread miswak usage compared to conventional products can provide insights into its sustainability and contribute to eco-friendly oral care practices.

## 5. Conclusion

Miswak (*Salvadora persica*) has demonstrated potential antibiofilm, antiplaque, and anticaries properties compared to conventional oral health measures. When *Salvadora persica* was used to maintain dental hygiene, there was a noteworthy decrease in the number of cariogenic bacteria and plaque. However, further research would establish its safety and efficacy, especially about long-term use and potential side effects. Additionally, the studies varied in methodology, sample size, and outcome measures, which limits the ability to draw definitive conclusions. The findings suggest that miswak could be a promising natural adjunct to conventional oral health measures in reducing dental biofilms and maintaining oral health.

## Figures and Tables

**Table 1:** Search strategy for all electronic databases.

PICO	Search strategy
Population	((((((((Dental caries[Title/Abstract]) OR (caries[Title/Abstract])) OR (decay[Title/Abstract])) OR (dental decay[Title/Abstract])) OR (carious teeth[Title/Abstract])) OR (carious lesions[Title/Abstract])) OR (dental disease[Title/Abstract])) OR (dental cavity[Title/Abstract])) OR (tooth decay[Title/Abstract])
Intervention	(((((((((((((((((((Salvadora persica[Title/Abstract]) OR (miswak[Title/Abstract])) OR (meswak[Title/Abstract])) OR (arak[Title/Abstract])) OR (pilu[Title/Abstract])) OR (siwak[Title/Abstract])) OR (salvadoraceae[Title/Abstract])) OR (herbal[Title/Abstract])) OR (toothbrush tree[Title/Abstract])) OR (chewing stick[Title/Abstract])) OR (toothbrush stick[Title/Abstract])) OR (natural toothbrush[Title/Abstract])) OR (herbal toothpaste[Title/Abstract])) OR (herbal tooth cream[Title/Abstract])) OR (tooth cleansing cream[Title/Abstract])) OR (salvadora persica toothpaste[Title/Abstract])) OR (salvadora persica mouthwash[Title/Abstract])) OR (miswak sticks[Title/Abstract])) OR (miswak twigs[Title/Abstract])) OR (miswak mouthwash[Title/Abstract])
Comparison	(((((((((((((toothpaste[Title/Abstract]) OR (toothbrush[Title/Abstract])) OR (fluoride[Title/Abstract])) OR (daily toothpaste[Title/Abstract])) OR (regular toothpaste[Title/Abstract])) OR (fluoridated tooth[Title/Abstract])) OR (fluoridated dental cream[Title/Abstract])) OR (dental hygiene[Title/Abstract])) OR (daily oral hygiene[Title/Abstract])) OR (mouthwash[Title/Abstract])) OR (mouth rinse[Title/Abstract])) OR (dental floss[Title/Abstract])) OR (tooth brushing[Title/Abstract])) OR (interdental aids[Title/Abstract])
Outcome	(((((((((((((Anticaries[Title/Abstract]) OR (Anti-cariogenic[Title/Abstract])) OR (decay[Title/Abstract])) OR (biofilm[Title/Abstract])) OR (antibacterial[Title/Abstract])) OR (antimicrobial[Title/Abstract])) OR (dental plaque[Title/Abstract])) OR (microbial load[Title/Abstract])) OR (antiplaque[Title/Abstract])) OR (cariogenic[Title/Abstract])) OR (anti-decay[Title/Abstract])) OR (bacterial count[Title/Abstract])) OR (bacterial load[Title/Abstract])) OR (microbial count[Title/Abstract])

**Table 2:** Excluded studies.

Title of the study	Authors	Year of publication	Reason for exclusion
Comparative Effectiveness of Chewing Stick and Toothbrush: A Randomized Clinical Trial	Aeeza S. Malik, Malik S. Shaukat, Ambrina A. Qureshi, Rasheed Abdur	2014	Neem was used instead of miswak as a primary intervention.
Comparison of Fluoridated Miswak and Toothbrushing With Fluoridated Toothpaste on Plaque Removal and Fluoride Release	Hosam Baeshen, Sabin Salahuddin, Robel Dam, Khalid H. Zawawi, Dowen Birkhed	2017	The concentration of fluoride alone was assessed.
Distribution of Dental Plaque and Gingivitis Within the Dental Arches	Prem K. Sreenivasan and Kakarla V. V. Prasad	2017	Miswak was not used as an intervention.

**Table 3:** Characteristics of the included studies.

Study reference	Location of the study	Study design	Population			Number of groups	Intervention		Comparison		Collection of samples	Outcomes assessed				Follow-up period
			Sample size	Age	Gender		Type of miswak form	Frequency of use	Control group	Frequency of use		Clinical parameters assessing decay	Clinical parameters assessing plaque	Bacterial count	Other parameters	
Al-Otaibi et al. 2003 [30]	Al-Noor Specialist Hospital, Saudi Arabia	Single blind, randomized cross-over design	15	21–36 years	Male: 15	2 groups: miswak + toothbrush, miswak alone	Fresh miswak chewing sticks	5 times a day	Toothbrushing (without toothpaste)	2 times a day	x	x	Turesky Modified Quigley–Hein plaque index	x	Loe–Silness gingival index (GI)	4 weeks
Al-Otaibi et al. 2004 [31]	Al-Noor Specialist Hospital, Saudi Arabia	Single blind, randomized cross-over design	15	21–36 years	Male: 15	2 groups: miswak + toothbrush, miswak alone	Fresh miswak chewing sticks	5 times a day	Toothbrushing (without toothpaste)	2 times a day	Plaque collection with paper points	x	Checkerboard DNA-DNA hybridization	Agar plate inhibition zone with *Actinobacillus actinomycetemcomitans*	x	4 weeks
Sofrata et al. 2011 [32]	Security Forces Dental Polyclinics, Saudi Arabia	Double-blinded, randomized controlled trial—parallel design	68	≥ 18 years	Male: 21 Female: 47	2 groups: fresh and inactive miswak	Fresh miswak sticks	5 times a day	Inactivate miswak sticks (deactivated by boiling for 2 h)	5 times a day	x	x	Modified Quigley–Hein plaque index	x	Modified gingival index (GI)	3 weeks
Patel et al. 2012 [33]		Single blind, randomized parallel design	30	18–35 years	Not mentioned	3 groups: toothbrush alone, toothbrush + miswak, miswak alone	Miswak twigs (refrigerated)	3 times a day	Toothbrushing	3 times a day	x	x	Turesky Modified Quigley–Hein plaque index	x	Loe–Silness gingival index (GI)	8 weeks
Al-Dabbagh et al. 2016 [34]	Zakho city, Kurdistan, Iraq	Randomized controlled trial	40	16–18 years	Male: 20 Female: 20	4 groups: miswak toothpaste miswak mouthwash ordinary toothpaste normal saline	Miswak toothpaste (Siwak.F, Saudia Arabia) miswak mouthwash (G, R. Lane Health Products Ltd, UK)	2 times a day	Ordinary toothpaste (Doctor Toothpaste, China) normal saline	2 times a day	Stimulated saliva	x	x	Colony forming units (CFU) count (*Streptococcus mutans*, Lactobacilli)	x	2 weeks
Raina et al. 2017 [35]	Krishnadevaraya College of Dental Sciences and Hospital, India	Triple-blinded, randomized controlled trial—parallel design	30	20–23 years	Male: 11 Female: 19	2 groups: fluoridated miswak and plain miswak sticks	Fluoridated miswak sticks (impregnated with 0.5% NaF)	6 min	Plain miswak sticks	6 min	Unstimulated saliva	x	x	Colony forming units (CFU) count (*Streptococcus mutans*)	x	No follow-up
Zarabadipour et al. 2020 [36]	Qazvin University of Medical Sciences, Iran	Randomized controlled trial	80	≥ 18 years	Male: 40 Female: 40	4 groups: Active siwak Inactive siwak Normal toothbrush No oral hygiene	Active and inactive siwak (miswak)	2 times a day	Toothbrush (Oral-B, Malaysia) No mechanical or chemical aids for oral hygiene	2 times a day	x	x	Loe and Silness plaque index	x	x	6 days
Ibrahim et al. 2022 [37]	University, Cairo, Egypt	Single-blinded, randomized controlled trial—parallel design	78	18–25 years	Male: 45 Female: 33	3 groups: Standard oral hygiene measures miswak only miswak plus toothbrush and toothpaste	Miswak alone miswak with toothbrush and toothpaste	2 times a day	Standard oral hygiene measures (includes toothbrush, toothpaste, dental floss, and mouth rinse)	2 times a day	x	DMF score	Plaque index (PI)	x	x	3, 6, 9, 12 months
Shaalan and El-Rashidy 2023 [38]	Faculty of Dentistry, Cairo University, Cairo, Egypt	Randomized clinical trial—parallel design	32	Mean age: 21.3 ± 2.5 years	Male: 14 Female: 18	2 groups: miswak toothpaste Fluoride toothpaste	Miswak toothpaste (Dabur miswak toothpaste)	2 times a day	Fluoridated toothpaste 1450 ppm (Signal Cavity Fighter)	2 times a day	Unstimulated saliva	x	x	Colony forming units (CFU) count (*Streptococcus mutans*)	x	1 week, 1, 3 months
Kengadaran et al. 2023 [39]	Saveetha Dental College and Hospitals, Chennai, India	Double-blinded, randomized controlled trial—parallel design	54	18–25 years	Male: 33.7% (18) Female: 66.7% (36)	3 groups: Herbal dentifrice homeopathic dentifrice fluoride dentifrice	Herbal dentifrice (Dabur meswak) homeopathic dentifrice (Gum Forte gel)	2 times a day	Fluoride dentifrice (Colgate Total)	2 times a day	Unstimulated saliva, supragingival plaque	x	x	Colony forming units (CFU) count (*Streptococcus mutans*, Lactobacilli)	pH of plaque and saliva	14, 28 days

## Data Availability

The datasets used and/or analyzed during the current study are available from the corresponding author on reasonable request.
